# Effect of rs67085638 in long non‐coding RNA (CCAT1) on colon cancer chemoresistance to paclitaxel through modulating the microRNA‐24‐3p and FSCN1

**DOI:** 10.1111/jcmm.16210

**Published:** 2021-03-11

**Authors:** Zhong‐Sheng Xiao, Lei Zhao, Xiao‐Ning Zhang, Han‐Xian Li, Zhi‐Hui Yin

**Affiliations:** ^1^ Department of Gastrointestinal Surgery The First Affiliated Hospital of University of South China Hengyang China; ^2^ Department of Anorectal Disease The First Affiliated Hospital of University of South China Hengyang China

**Keywords:** CCAT1, colon cancer, FSCN1, miR‐24‐3p, paclitaxel, SNP

## Abstract

It has been reported that rs67085638 in long non‐coding RNAs (lncRNA)‐CCAT1 was associated with the risk of tumorigenesis. Also, CCAT1 could affect chemoresistance of cancer cells to paclitaxel (PTX) via regulating miR‐24‐3p and FSCN1 expression. In this study, we aimed to investigate the effect of rs67085638 on the expression of CCAT1/miR‐24‐3p/FSCN1 and the response of colon cancer to the treatment of PTX. 48 colon cancer patients were recruited and grouped by their genotypes of rs67085638 polymorphism as a CC group (N = 28) and a CT group (N = 20). PCR analysis, IHC assay and Western blot, TUNEL assay and flow cytometry were conducted. LncRNA‐CCAT1 and FSCN1 mRNA/protein were overexpressed, whereas miR‐24‐3p was down‐regulated in the CT‐genotyped patients and cells compared with those in the CC‐genotyped patients and cells. The survival of colon cancer cells was decreased, whereas the apoptosis of colon cancer cells was increased by PTX treatment in a dose‐dependent manner. MiR‐24‐3p was validated to target lncRNA‐CCAT1 and FSCN1 mRNA, and the overexpression of CCAT1 could reduce the expression of miR‐24‐3p although elevating the expression of FSCN1. Knockdown of lncRNA‐CCAT1 partly reversed the suppressed growth of CT‐genotyped tumours. And the knockdown of lncRNA‐CCAT1 partly reversed the dysregulation of lncRNA‐CCAT1 and FSCN1 mRNA/protein in rs67085638‐CT + NC shRNA mice. The findings of this study demonstrated that the presence of the minor allele of rs67085638 increased the expression of CCAT1 and accordingly enhanced the resistance to PTX. Down‐regulation of CCAT1 significantly re‐stored the sensitivity to PTX of colon cancer cells.

## 1 INTRODUCTION

Colon cancer is a type of common malignant tumours in the digestion system that occurs mainly at the junction of the sigmoid colon and rectum, with the greatest incidence in people of 40 to 50 years old.^1^ Colon cancer attributes to 1/3 of all malignant tumour cases worldwide. Colon cancer is mainly classified as undifferentiated carcinoma, mucinous adenocarcinoma and adenocarcinoma. The general form of colon cancer is ulcers or polypoid.^2^ Individuals with colon polyps or persistent colitis as well as obese males are predominantly vulnerable to colon cancer.^3^ Although non‐specific cytotoxicity reduces the therapeutic index of colon cancer, causing small differences in treatment efficacy, the resistance to 5‐FU treatment is commonly seen to result in a poor prognosis.^4^

With its broad spectrum anti‐tumour functions as well as its capacity to prevent angiogenic features of endothelial cells, paclitaxel seems like a strong prospective agent for chemotherapy.^5^, ^6^ All current results showed that the mix of a low‐dose therapy of paclitaxel (20 mg/kg in mice models) with the knockdown of hypoxia‐inducible factor‐1a may induce a potent inhibitory effect against colon cancer cells.^7^

Long non‐coding RNAs (lncRNAs) are a type of non‐coding RNAs containing more than 200 nucleotides. LncRNAs were reported to play essential roles as epigenetic mediators of gene expression.^8^, ^9^ More evidence indicated that the dysregulated lncRNA expression is associated with the pathogenesis of various disorders such as cancers.^10^ The polymorphisms in lncRNAs may show numerous impacts on the expression as well as functions of lncRNAs, therefore regulating the cancer susceptibility of individuals.^11^ Colorectal cancer associated transcript 1 (CCAT1) is an lncRNA of about 11 kb, and the overexpression of CCAT1 appears in both the tumorigenesis and progression of CRC.^12^ Xiang et al actually showed that CCAT1 played a key role in the transcriptional regulation of MYC as well as in promoting chromatin looping. The knockdown of CCAT1 decreases the interaction between the promoter of MYC with its enhancer.^13^ Cell growth as well as MYC transcription is strongly correlated with the expression of CCAT1 mRNA in multiple types of tumours.^14^

MiR‐24‐3p is an miRNA whose role has been ignored in cardiac ischaemia/reperfusion injury (I/RI). In the last few years, miR‐24‐3p has been extensively studied as a tumour suppressor in many types of tumours, including nasopharyngeal carcinoma, liver cancer and lung cancer, indicating that miR‐24‐3p contributes in the process of tumour growth suppression as well as enhancing the apoptosis of tumour cells.^15^, ^16^ It was actually presented that the expression of miR‐24‐3p was negatively moderated via CCAT1, whereas the depletion of miR‐24‐3p eliminated the suppressive impact of knockdown of CCAT1 on PTX resistance, suggesting that CCAT1 managed PTX resistance through sponging the expression of miR‐24‐3p.^17^ Li et al have shown that miR‐24 could reduce the migration, proliferation, invasion and growth of tumour cells through targeting the FSCN1 expression in nasopharyngeal cancer.^17^

As an actin‐binding protein with a molecular weight of 55 kD, FSCN1 works as a significant factor in keeping the stability of filamentous actins. FSCN1 arranges F‐actins in cells to create tightly packed bundles of F‐actins to assist the formation of certain primary structures based on actin: Cortical cell projections that moderate cell‐cell communications and cell migration, as well as cytoplasmic microfilaments that aid the formation of cell structures and intracellular movement.^18^, ^19^ FSCN1 has recently become a focus of research as a novel biomarker for many types of cancers. FSCN1 showed reduced expression in normal epithelial cells, but its expression is increased in oesophageal cancer, lung cancer, breast cancer and prostate cancer.^18^, ^20^

It has been reported that a single nucleotide polymorphism (rs67085638) in lncRNA‐CCAT1 was associated with the risk of tumorigenesis and rs67085638 C > T SNP in lncRNA CCAT1 was linked to an enhanced risk of colon cancer, and SNP rs6983267 was also shown to be situated in the MYC enhancer region plays a role in the regulation of CCAT1 expression.^21^ Another study showed that CCAT1 may affect the chemoresistance of ovarian cancer cells to PTX via regulating miR‐24‐3p and FSCN1.^17^ In this study, we investigated the effect of rs67085638 on the expression of CCAT1/miR‐24‐3p/FSCN1 and the response of colon cancer to the treatment of PTX.

## 2 MATERIALS AND METHODS

### 2.1 Patient recruitment

In this study, we recruited 48 colon cancer patients seeking treatment at our hospital during September 2016 to March 2018. The tumour tissues of these patients were collected for genotyping of the rs67085638 polymorphism (see below). Based on the results of genotyping of the rs67085638 polymorphism, the patients were grouped into a CC group (N = 28) and a CT group (N = 20). The demographic and clinicopathological characteristics of each patient were also collected and compared between the two patient groups. Institutional ethical committee has approved the study.

### 2.2 Animal model

Cancerous colon cells were collected from the colon cancer patients genotyped as CC or CT. Then, three cell groups were set up, that is an rs67085638‐CC group (cancerous colon cells genotyped as CC), an rs67085638‐CT + NC shRNA group (cancerous colon cells genotyped as CT and then treated with NC shRNA) and an rs67085638‐CT + shRNA‐CCAT1 group (cancerous colon cells genotyped as CT and then treated with shRNA against lncRNA‐CCAT1).

Accordingly, 4‐week old BALB/c male nude mice with a bodyweight of 19 ± 1 g were purchased from the animal facility of our hospital. This research study was approved by the Ethics Committee of our hospital. The mice were given unlimited access to a standard pellet diet as well as unlimited drinking water. The mice were placed in an airy room with a regulated temperature of 25 ± 2˚C and a 12‐hour/12‐hour dark/light cycle. After 7 days of adaptation, the mice were arbitrarily divided in 3 groups corresponding to the three groups of cells described above (n = 10 in each group), that is 1. rs67085638‐CC mice; 2. rs67085638‐CT + NC shRNA mice; and 3. rs67085638‐CT + shRNA‐CCAT1 mice. Subsequently, to generate a mouse model of colon cancer, the cells described above were administered via subcutaneous injection into the rear side of each mouse, which was then treated with PTX at a dose of 12 mg/kg injected via the intravenous injection through the tail every three days for a total of 21 days to study the expression of target genes. After 21 days of treatment, the mice were killed through deep anaesthesia and the tumour in each mouse was removed to measure its weight and volume by making use of the formula: V = (W × W × L)/2, where V was the tumour volume, W was the tumour width, whereas L was the tumour length.

### 2.3 Cell culture and transfection

Primary colon cancer cells were isolated from colon cancer patients and maintained in a modified Dulbecco's Eagle medium (DMEM) supplemented with 10% of foetal bovine serum and antibiotics of trypsin and streptomycin (Gibco, Thermo Fisher Scientific). Then, the cells were divided into 3 groups, that is (a) rs67085638‐CC group; (b) rs67085638‐CT + NC shRNA; and (c) rs67085638‐CT + shRNA‐CCAT1. In the next step, the cells in groups rs67085638‐CT + NC shRNA and rs67085638‐CT + shRNA‐CCAT1 were transfected with corresponding shRNAs using Lipofectamine 2000 (Invitrogen) according to the instructions provided by the manufacturer. At 48 hours after transfection, the cells were collected to assay the expression of target genes. In addition, some of the cells were treated with different concentrations of PTX, and the survival status of the cells was measured using an MTT assay. In brief, primary colon cancer cells were treated with different concentrations of PTX for two days. Then, an MTT assay kit bought from Aladdin (Shanghai, China) was used according to the instructions provided by the manufacturer. Ultimately, the absorbance in each well was read at a wavelength of 490 nm by utilizing a microplate reader (Bio‐Rad, Hercules) to plot the survival curve and calculate the survival of cells at different time‐points, thus calculating the IC50 of PTX.

### 2.4 RNA isolation and real‐time PCR

Collected tumour tissues and cell samples were treated with TRIzol (Invitrogen) according to the instructions provided by the manufacturer to extract total RNA of each sample. Then, a QuantScript reverse transcription assay kit (Tiangen Biotech) was used for 60 minutes of reverse transcription at 37°C to obtain cDNA, which was subjected to real‐time PCR by using a Tiangen qPCR assay kit (Tiangen Biotech) according to the instructions provided by the manufacturer. The real‐time PCR reactions were carried out in a Step OnePlus Real‐Time PCR machine. Finally, the relative expression of lncRNA‐CCAT1, miR‐24‐3p and FSCN1 mRNA in each sample was calculated by using the 2^−ΔΔCt^ approach. U6 and 146 GAPDH were utilized as the housekeeping gene for the normalization. The primer pairs used in this study were as follows: Forward: 5’‐CATTGGGAAAGGTG‐CCGAGA‐3’ and Reverse: 5’‐ACGCTTAGCCATACAGAGCC‐3’ for lncRNA‐CCAT1; Forward: 5’‐GCCTACTGAGCTGATATC‐3’ and Reverse: 5’‐ GAACATGTCTGCGTATCTC‐3’ for miR‐24‐3p; Forward: 5’‐GACACCAAAAAGTGTGCCTTCCG‐3’ and Reverse: 5’‐CAAACTTGCCATTGGACGCCCT‐3’ for FSCN1 mRNA; Forward: 5’‐GTGCTCGCTTCGGCAGCA‐3’ and Reverse: 5’‐CAAAATATGGAACGCTTC‐3’ for U6; Forward: 5’‐GGGAGCCAAAAGG GTCAT‐3’ and Reverse: 5’‐GAGTCCTTCCACGATACCAA‐3’ for GAPDH.

### 2.5 Luciferase assay

Online bioinformatic tools including TargetScan and Pictar‐Vert were used to predict the binding sites of miR‐24‐3p on FSCN1 mRNA and lncRNA‐CCAT1. Then, wild‐type lncRNA‐CCAT1 sequence and 3’ UTR of FSCN1 mRNA containing the miR‐24‐3p binding sites were, respectively, cloned into pcDNA3.1 vectors (Promega) to create wild‐type lncRNA‐CCAT1 and FSCN1 mRNA vectors. At the same time, a Quick Change mutagenesis kit (Stratagene) was used to create site directed mutations in the miR‐24 binding sites of lncRNA‐CCAT1 and FSCN1 mRNA, and the mutated sequences were also respectively cloned into pcDNA3.1 vectors to create mutant type lncRNA‐CCAT1 and FSCN1 mRNA vectors. Finally, CACO‐2 cells were co‐transfected with lncRNA‐CCAT1 and miR‐24‐3p mimics or FSCN1 mRNA and miR‐24 −3p mimics, and a dual‐luciferase reporter gene assay kit (Promega) was used 24 hours later according to the instructions provided by the manufacturer to determine the luciferase activity of transfected cells.

### 2.6 Survival rate

The rates of apoptosis and survival were determined with FCM analysis by making use of an Annexin V‐APC/PI Apoptosis Assay kit (KeyGen Biotech) according to the instructions provided by the manufacturer. The reading of results was carried out on a FACS Canto II flow cytometer (BD Biosciences) utilizing the FlowJo X.10.0.7‐1 software (Bd Biosciences).

### 2.7 Western blot analysis

The expression level of FSCN1 in each sample was determined through Western blotting. In brief, the samples were lysed in a radioimmunoprecipitation buffer to extract total protein, which was then resolved on 10% SDS‐PAGE. In the next step, the resolved proteins were electrophoretically blotted onto polyvinylidene fluoride (PVDF) membranes, which were then blocked with 5% skim milk at ambient temperature for 1 hour. Then, the membranes were incubated along with anti‐FSCN1 (1:1000, Abcam) primary antibodies and HRP‐labelled secondary antibodies under conditions suggested by the antibody manufacturer. Finally, the membranes were developed in an enhanced chemiluminescence (ECL) reagent (Thermo Fisher Scientific) and the densitometry analysis of protein bands was performed using ImageJ software to determine the relative protein expression of FSCN1 in each sample.

### 2.8 Immunohistochemical (IHC) assay

The collected tissue samples were fixed by using 10% neutral formalin (Thermo Fisher Scientific) according to the instructions provided by the manufacturer. After embedding in paraffin and partitioning into 5 µm slices, the tissue sections were dewaxed, rehydrated and subjected to antigen retrieval. Then, the slides were blocked by using bovine albumin and treated with anti‐FSCN1 primary antibodies and biotin labelled secondary antibodies under conditions suggested by the antibody manufacturer. Finally, the slides were observed under a fluorescence microscope to determine the positive expression rate of FSCN1 in each sample.

### 2.9 TUNEL assay

Cell apoptosis was analysed by using a terminal deoxynucleotidyl transferase‐mediated X‐dUTP nick end labelling (TUNEL) assay kit (Roche) according to the instructions provided by the manufacturer.

### 2.10 Statistical analysis

All statistical analyses were performed by utilizing GraphPad Prism 7.0 software (GraphPad). All results were presented as the mean ± standard deviation (SD, represented by error bar). Comparisons of two different groups were accomplished by making use of Student's t tests, and comparisons of multiple groups were accomplished by one‑way ANOVA with Bonferroni's test being utilized as a post hoc test. *P* < .05 was taken into consideration to show a statistically significant difference.

## 3 RESULTS

### 3.1 Colon cancer patient recruitment

In this study, we recruited 48 colon cancer patients and grouped them by their genotypes of the rs67085638 polymorphism as a CC group (N = 28) and a CT group (N = 20). We measured the demographic and clinicopathological characteristics including age, gender, drinking habits, smoking habits and BMI of each patient and collected and demonstrated the results in Table 1. Accordingly, between the CC and CT patient group, we found no evident differences of these characteristics among these two patient groups in respect to the age, gender, drinking habits or smoking habits. Therefore, it can be suggested that the genotypes of rs67085638 polymorphism were not affected by or correlated with these characteristics.

**TABLE 1:** Demographic and clinicopathological characteristics of colon cancer patients grouped according to their genotypes of rs67085638

Characteristics	Serum circHIPK3		*P* value
	CC (N = 28)	CT (N = 20)	
Age (y)	52.6 ± 7.0	53.3 ± 6.6	.137
Gender			
Male	15 (53.6)	8 (40.0)	.357
Female	13 (46.4)	12 (60.0)	
Alcohol drinking			
Yes	6 (21.4)	3 (15.0)	.546
No	14 (78.6)	17 (85.0)	
Smoking			
Yes	6 (21.4)	5 (25.0)	.404
No	14 (78.6)	17 (75.0)	
BMI (kg/m^2^)	24.7 ± 2.1	24.9 ± 2.9	24.5 ± 3.1

### 3.2 Gene expression in colon cancer patients

**FIGURE 1:**
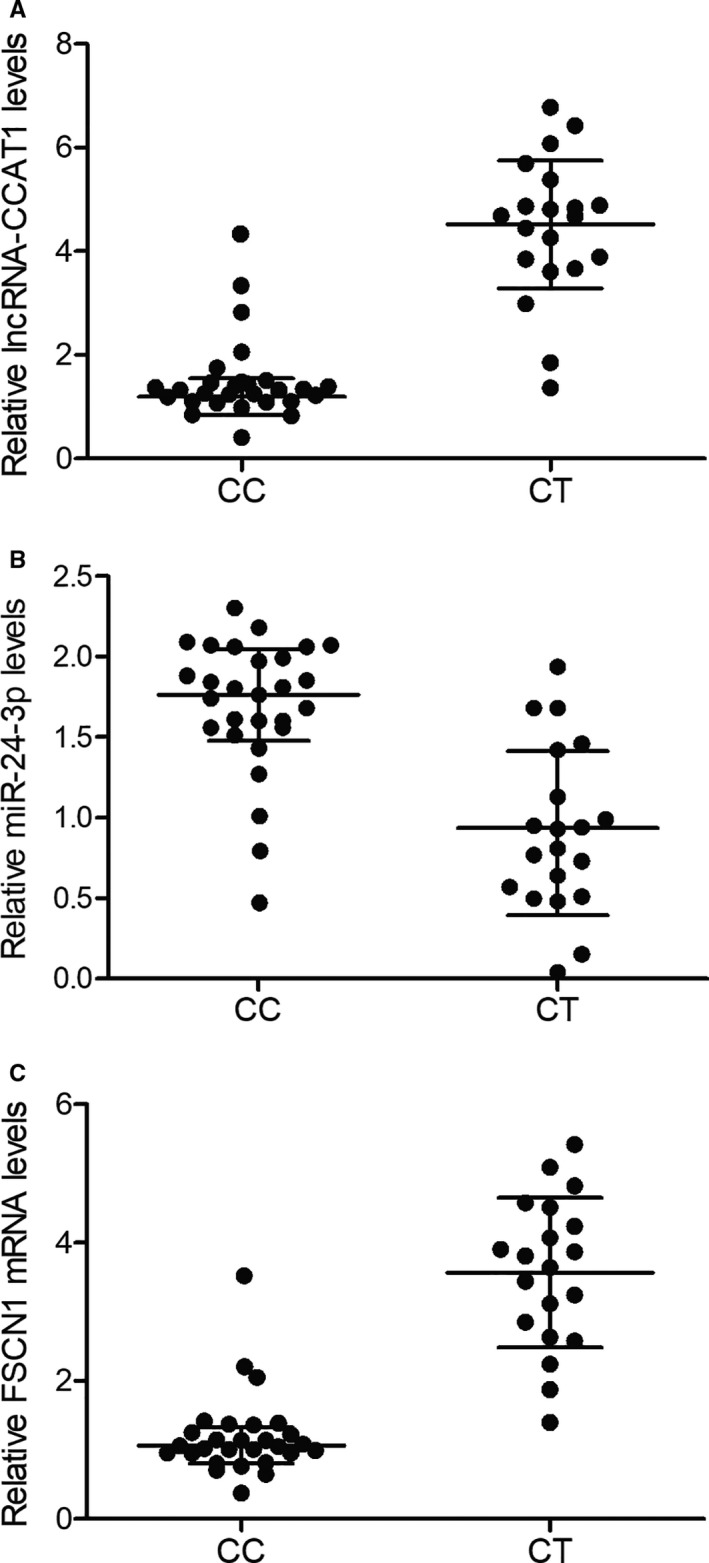
Expression of lncRNA‐CCAT1, miR‐24‐3p and FSCN1 mRNA in colon cancer patients with different genotypes. A, The expression of lncRNA‐CCAT1 in the CC group and the CT group. B, The expression of miR‐24‐3p in the CC group and the CT group. C, The expression of FSCN1 mRNA in the CC group and the CT group

**FIGURE 2:**
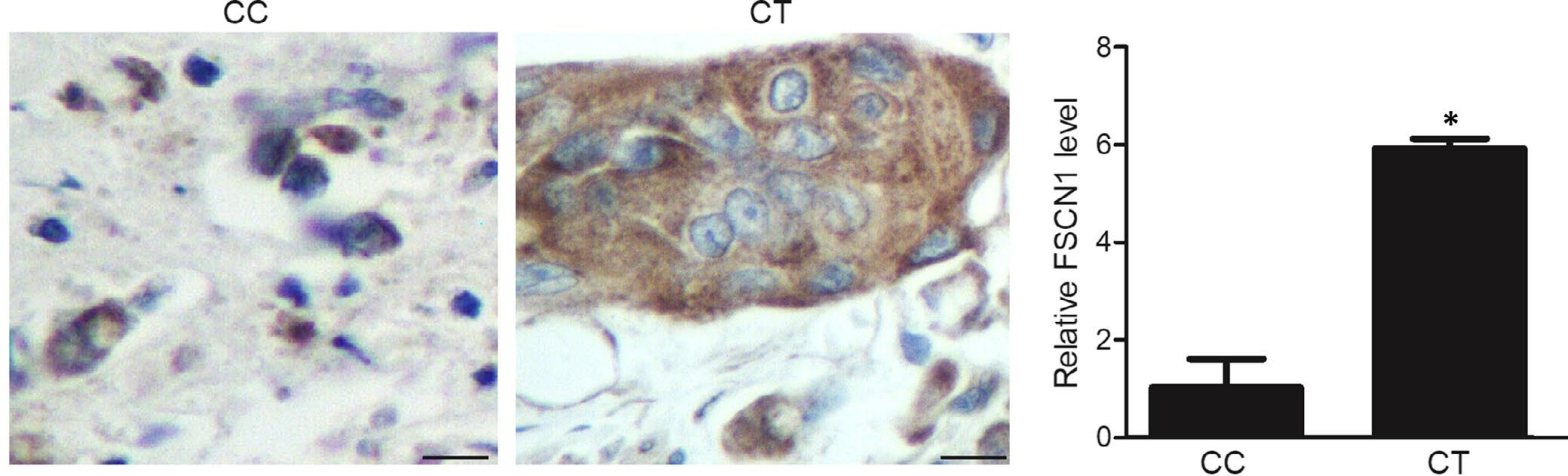
IHC assay indicated the expression of FSCN1 protein in colon cancer patients with CC and CT genotypes (^*^ *P*‐value < .05 compared with CC group)

Expression of target genes of this study, including lncRNA‐CCAT1, miR‐24‐3p and FSCN1 mRNA, was observed in the cancerous tissue samples. Accordingly, compared with the CC‐genotyped patients, the CT‐genotyped patients showed markedly up‐regulated expression of lncRNA‐CCAT1 (Figure 1A) and FSCN1 mRNA (Figure 1C) along with the evidently down‐regulated expression of miR‐24‐3p (Figure 1B). Moreover, we observed an evident increase of FSCN1 protein (Figure 2) expression in the CT group compared with that in the CC group.

### 3.3 Gene expression in colon cancer cells of different genotypes

**FIGURE 3:**
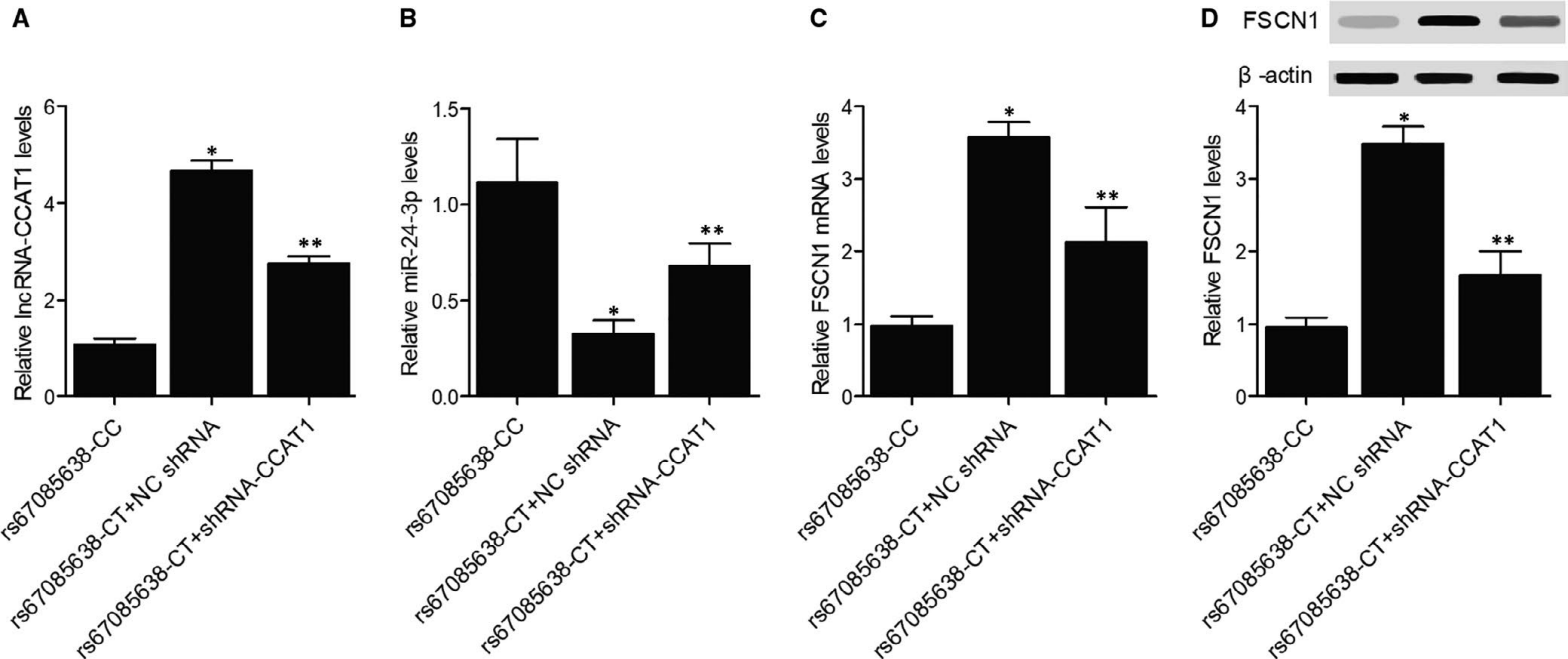
Expression of lncRNA‐CCAT1, miR‐24‐3p and FSCN1 mRNA in colon cancer cells collected from patients with different genotypes (^*^ *P*‐value < .05 compared with rs67085638‐CC group; ^**^ *P*‐value < .05 compared with rs67085638‐CT + NC shRNA group). A, The expression of lncRNA‐CCAT1 in the rs67085638‐CC group, the rs67085638‐CT + NC shRNA group and the rs67085638‐CT + shRNA‐CCAT1 group. B, The expression of miR‐24‐3p in the rs67085638‐CC group, the rs67085638‐CT + NC shRNA group and the rs67085638‐CT + shRNA‐CCAT1 group. C, The expression of FSCN1 mRNA in the rs67085638‐CC group, the rs67085638‐CT + NC shRNA group and the rs67085638‐CT + shRNA‐CCAT1 group. D: The expression of FSCN1 protein in the rs67085638‐CC group, the rs67085638‐CT + NC shRNA group and the rs67085638‐CT + shRNA‐CCAT1 group

Cancerous colon cells collected from the colon cancer patients genotyped as CC or CT were collected. Three cell groups were set up. Compared with the rs67085638‐CC group, we observed an evident increase of lncRNA‐CCAT1 (Figure 3A) and FSCN1 mRNA (Figure 3C) expression, as well as a significant decrease of miR‐24‐3p (Figure 3B) expression, in the rs67085638‐CT + shRNA‐CCAT1 group. And the knockdown of lncRNA‐CCAT1 by CCAT1 shRNA resulted in suppressed lncRNA‐CCAT1 (Figure 3A) and FSCN1 mRNA (Figure 3C) expression along with increased miR‐24‐3p (Figure 3B) expression in CT‐genotyped cells. Also, Western blot analysis validated the changes of FSCN1 protein (Figure 3D) expression in different groups of colon cancer cells.

### 3.4 Effects of PTX treatment on the survival and apoptosis of colon cancer cells

**FIGURE 4:**
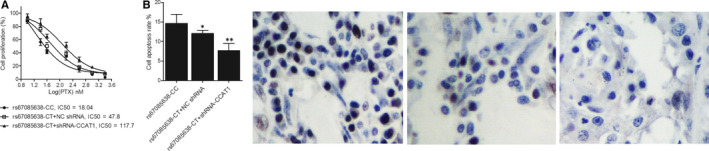
Survival and apoptosis induced by PTX treatment of colon cancer cells collected from patients with different genotypes (^*^ *P*‐value < .05 compared with rs67085638‐CC group; ^**^ *P*‐value < .05 compared with rs67085638‐CT + NC shRNA group). A, The survival rate of the rs67085638‐CC group, the rs67085638‐CT + NC shRNA group and the rs67085638‐CT + shRNA‐CCAT1 group treated with PTX at different concentrations. B, The TUNEL assay representative results and according calculated apoptosis rate of the rs67085638‐CC group, the rs67085638‐CT + NC shRNA group and the rs67085638‐CT + shRNA‐CCAT1 group treated with PTX at different concentrations

The colon cancer cells of different genotypes were subjected to PTX treatment at concentrations of 1 nmol/L, 1.4 nmol/L, 1.8 nmol/L, 2.2 nmol/L, 2.6 nmol/L, 3.0 nmol/L and 3.4 nmol/L. The survival and apoptosis rates of each cell group were measured by flow cytometry, respectively. As shown in Figure 4A, the survival of each cell group was decreased by the PTX treatment in a dose‐dependent manner. However, the survival rate of CT‐genotyped cells was higher than that of the CC‐genotyped cells, whereas the knockdown of lncRNA‐CCAT1 in CT‐genotyped cells suppressed their survival. On the contrary, as shown in Figure 4B, the apoptosis rate calculated from TUNEL assay results of CT‐genotyped cells was lower than that of the CC‐genotyped cells. Additionally, the survival rate of the rs67085638‐CT + NC shRNA group was higher than that of the rs67085638‐CT + shRNA‐CCAT1 group.

### 3.5 Establishment of a CCAT1/miR‐24‐3p/FSCN1 signalling pathway

**FIGURE 5:**
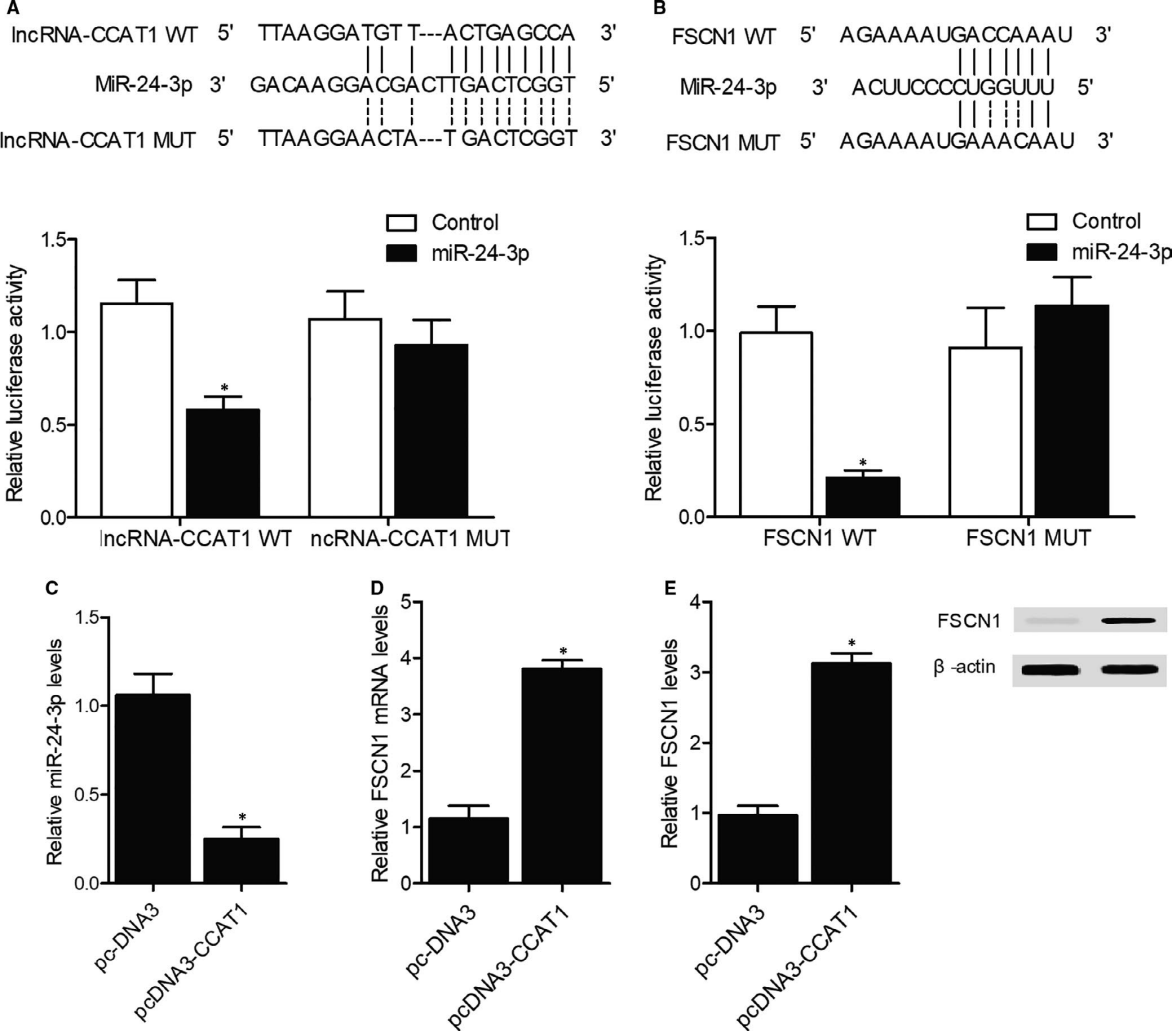
The CCAT1/miR‐24‐3p/FSCN1 signal**l**ing pathway was established in CACO‐2 cells. A, Sequence analysis and luciferase assay of miR‐24‐3p and lncRNA‐CCAT1 (^*^ *P*‐value < .05 compared with lncRNA‐CCAT1 WT + control group). B, Sequence analysis and luciferase assay of miR‐24‐3p and FSCN1 3’UTR (^*^ *P*‐value < .05 compared with FSCN1 WT + control group). C, Expression of miR‐24‐3p in the pcDNA3 group and the pcDNA3‐CCAT1 group (^*^ *P*‐value < .05 compared with pcDNA3 group). D, Expression of FSCN1 mRNA in the pcDNA3 group and the pcDNA3‐CCAT1 group (^*^ *P*‐value < .05 compared with pcDNA3 group). E, Expression of FSCN1 protein in the pcDNA3 group and the pcDNA3‐CCAT1 group (^*^ *P*‐value < .05 compared with pcDNA3 group)

Online bioinformatic tools including TargetScan and Pictar‐Vert were utilized to compare the sequences of lncRNA‐CCAT1, miR‐24‐3p and FSCN1 mRNA. Accordingly, as shown in Figure 5A, we detected a putative binding site of miR‐24‐3p on lncRNA‐CCAT1, and the subsequent luciferase assay showed markedly reduced luciferase activity of wild‐type lncRNA‐CCAT1 in CACO‐2 cells transfected with miR‐24‐3p mimics. Additionally, as shown in Figure 5B A binding site was also detected in the 3’UTR of FSCN1 mRNA, and the luciferase activity was significantly reduced in CACO‐2 cells co‐transfected with miR‐24‐3p and wild‐type FSCN1 3’UTR, indicating that miR‐24‐3p could target the expression of FSCN1 mRNA in CACO‐2 cells.

CACO‐2 cells were subsequently transfected with empty pcDNA3 vectors (pcDNA3 group) or pcDNA3 vectors carrying CCAT1 (pcDNA3‐CCAT1 group). As shown in Figure 5C, the expression of miR‐24‐3p was reduced (Figure 5C) whereas the expression of FSCN1 mRNA (Figure 5D) and protein (Figure 5E) was elevated in the pcDNA3‐CCAT1 group compared with that in the pcDNA3 group.

### 3.6 Xenograft study of colon tumours

**FIGURE 6:**
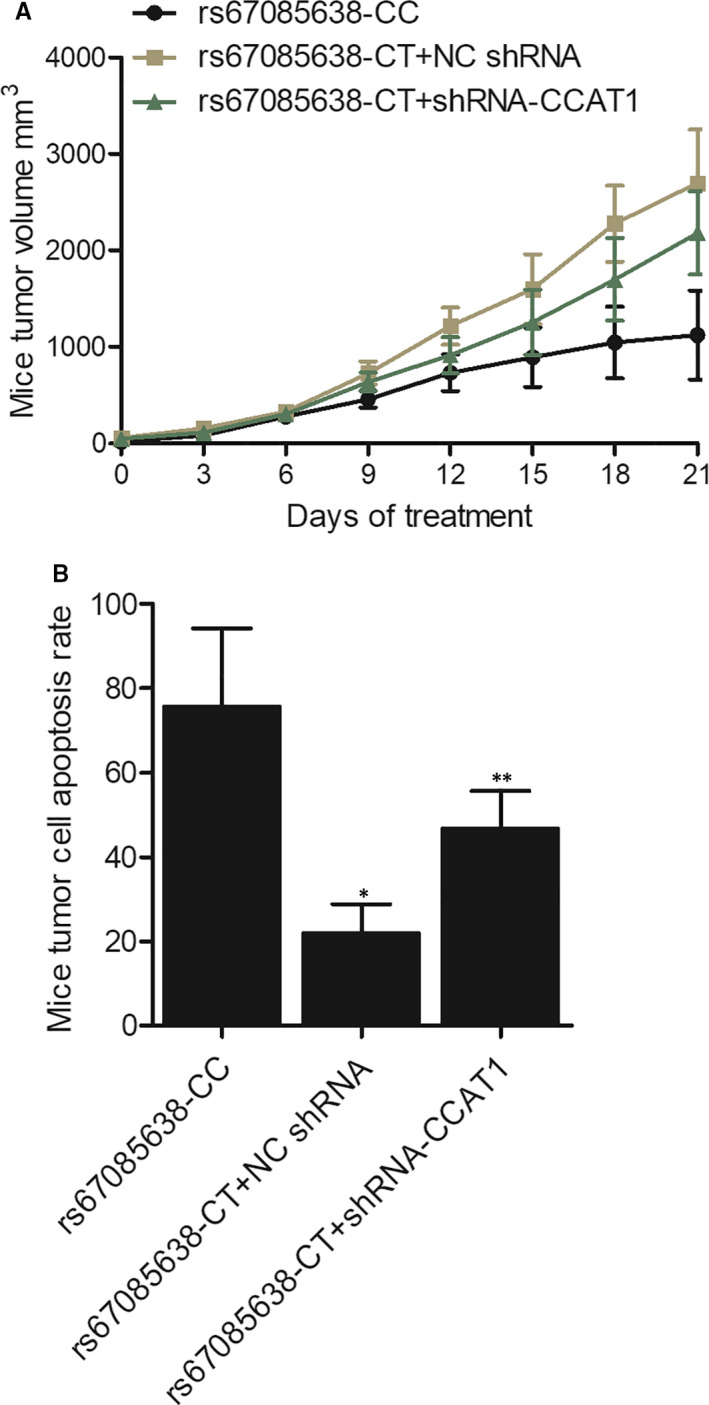
The tumour volume and the tumour cell apoptosis rate of the xenograft in the rs67085638‐CC mice, the rs67085638‐CT + NC shRNA mice and the rs67085638‐CT + shRNA‐CCAT1 mice (^*^ *P*‐value < .05 compared with rs67085638‐CC group; ^**^ *P*‐value < .05 compared with rs67085638‐CT + NC shRNA group). A, The tumour volume in the rs67085638‐CC mice, the rs67085638‐CT + NC shRNA mice and the rs67085638‐CT + shRNA‐CCAT1 mice. B, The tumour cell apoptosis rate in the rs67085638‐CC mice, the rs67085638‐CT + NC shRNA mice and the rs67085638‐CT + shRNA‐CCAT1 mice

The colon tumour cells were transplanted to mice to establish different groups of mice: rs67085638‐CC mice, rs67085638‐CT + NC shRNA mice and rs67085638‐CT + shRNA‐CCAT1 mice. The xenograft in each group of mice was studied, and the tumour volume and tumour cell apoptosis rate were measured. Accordingly, the mouse tumour volume was the highest in the rs67085638‐CT + NC shRNA mice and the lowest in the rs67085638‐CC mice (Figure 6A). Also, the tumour cell apoptosis rate was the highest in the rs67085638‐CC mice and the lowest in the rs67085638‐CT + NC shRNA mice (Figure 6B), thus validating that the tumour growth was more suppressed in CT‐genotyped tumours, whereas the knockdown of lncRNA‐CCAT1 partly reversed the effect of the CT genotype.

**FIGURE 7:**
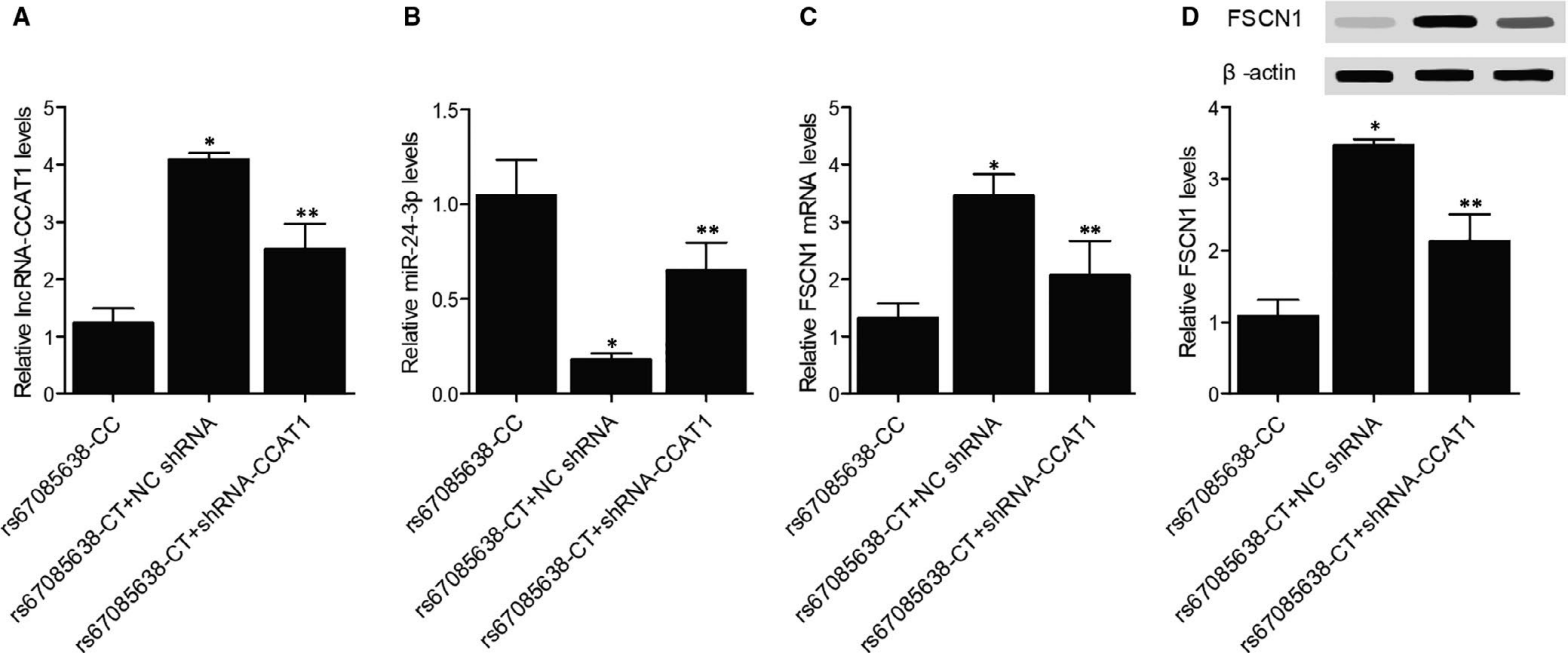
Expression of lncRNA‐CCAT1, miR‐24‐3p and FSCN1 mRNA in xenograft mice (^*^ *P*‐value < .05 compared with rs67085638‐CC group; ^**^ *P*‐value < .05 compared with rs67085638‐CT + NC shRNA group). A, The expression of lncRNA‐CCAT1 in the rs67085638‐CC mice, the rs67085638‐CT + NC shRNA mice and the rs67085638‐CT + shRNA‐CCAT1 mice. B, The expression of miR‐24‐3p in the rs67085638‐CC mice, the rs67085638‐CT + NC shRNA mice and the rs67085638‐CT + shRNA‐CCAT1 mice. C, The expression of FSCN1 mRNA in the rs67085638‐CC mice, the rs67085638‐CT + NC shRNA mice and the rs67085638‐CT + shRNA‐CCAT1 mice. D, The expression of FSCN1 protein in the rs67085638‐CC mice, the rs67085638‐CT + NC shRNA mice and the rs67085638‐CT + shRNA‐CCAT1 mice

Furthermore, compared with the rs67085638‐CC mice, the expression levels of lncRNA‐CCAT1 (Figure 7A) and FSCN1 mRNA (Figure 7C)/protein (Figure 7D) were significantly increased in the rs67085638‐CT + NC shRNA mice, and the knockdown of lncRNA‐CCAT1 partly reversed the dysregulation of lncRNA‐CCAT1 and FSCN1 mRNA/protein. On the contrary, as shown in Figure 7B, miR‐24‐3p was decreased in the rs67085638‐CT + NC shRNA and rs67085638‐CT + shRNA‐CCAT1 mice, with the rs67085638‐CT + NC shRNA mice showing the lowest level of miR‐24‐3p.

## 4 DISCUSSION

In this study, we collected tissue samples from OC patients who were sensitive or blunt to the treatment of PTX. We found that lncRNA‐CCAT1 and FSCN1 mRNA/protein were up‐regulated, whereas the level of miR‐24‐3p was down‐regulated in the CT group compared with that in the CC group. Furthermore, we also found that the survival of colon cancer cells was decreased whereas the apoptosis of colon cancer cells was increased by the PTX treatment in a dose‐dependent manner. However, the survival rate of CT‐genotyped cells was higher whereas the apoptosis rate of CT‐genotyped cells was lower than that of the CC‐genotyped cells, and the effects of the CC‐genotype were partly blocked by the knockdown of lncRNA‐CCAT1. It was revealed that the distribution of 3 SNPs, rs67085638, rs7013433 and rs77628730 in the CCAT1 gene was associated with the risk of CRC. However, even in the presence of such association, only the polymorphism of rs67085638 was discovered to be associated with an elevated CRC risk after adjusting for the age, education level, sex, BMI, as well as the ratio of red meat product and root vegetables in the diet. In fact, the polymorphism of rs67085638 was discovered to be associated with an elevated colon cancer risk but not an elevated rectal cancer risk.^21^ Another research showed in line results by revealing that the polymorphism of rs67085638 in the 3’UTR of CCAT1 was linked to elevated colon cancer and rectal cancer risks.^21^ One team of scientists presented that as compared to healthy cells, CCAT1 was confirmed to become overexpressed in colon tumour cells to enhance the invasion as well as proliferation of colon tumour cells. In fact, CCTA1 is actually associated with the metastasis of lymph nodes and patient prognosis.^22^ Sun et al showed that CCAT1 was a prospective biomarker of colonic tumours, which showed that CCAT1 may be made use of to estimate the prognosis of colon cancer.^23^ It was likewise shown that the IC50 value of PTX was reduced in PC3‐TXR cells than in DU145‐TXR cells, suggesting that DU145 cells displayed higher resistance to PTX.^24^ Significantly, the study on PTX resistance showed that the presence of CCAT1 lowered the IC50 value of PTX as well as cell viability yet boosted the apoptosis of both DU145‐TXR and PC3‐TXR cells.^25^ In this study, the CCAT1/miR‐24‐3p/FSCN1 signal**l**ing pathway was established by validating the targets of miR‐24‐3p. The overexpression of CCAT1 could reduce the expression of miR‐24‐3p whereas elevating the expression of FSCN1 mRNA and protein. In addition, we also found that, compared with the rs67085638‐CC group, the rs67085638‐CT + shRNA‐CCAT1 group showed an evident increase of lncRNA‐CCAT1 and FSCN1 mRNA/protein expression and a significant decrease of miR‐24‐3p expression, and the effects of the CC‐genotype were partly blocked by the knockdown of lncRNA‐CCAT1.

Previous research revealed that the prospective downstream targets of CCAT1 included miR‐24‐3p, which may bind to CCAT1 in RNA pull‐down, RIP and luciferase assays.^17^

The bioinformatics evaluations illustrated the prospective binding site of miR‐24‐3p in FSCN1. Subsequently, the presence of miR‐24‐3p caused a reduction of luciferase activity in both DU145‐TXR and PC3‐TXR cells. In addition, miR‐24‐3p overexpression led to even more FSCN1 expression in the cells.^17^ Gao et al^18^ displayed that miR‐24‐3p was actually down‐regulated in CC cells, but the miR‐24‐3p overexpression suppressed the proliferation of CC cells. Mishra et al displayed that miR‐24 acts as a p53‐independent tumour suppressor by suppressing the expression of dihydrofolate reductase in CC cells.^26^ Moreover, Fang et al revealed that the blood level of miR‐24 was reduced in CC patients.^27^

Moreover, it was shown that the expression of miR‐24‐3p was actually increased with the reduced expression of CCAT1 to eliminate the suppressive impact of CCAT1 on PTX resistance, suggesting that CCAT1 controlled PTX resistance through sponging the expression of miR‐24‐3p.^17^

As an evolutionarily preserved actin‐binding protein in the filopodia, Fascin1 is associated with cell migration using actin‐based protrusions located below the cell membrane.^19^, ^28^ Fascin1 participates in cell migration, adhesion and invasion in the bowel cancer as well as melanoma and is associated with poor cancer prognosis.^29^, ^30^, ^31^, ^32^ Additionally, FSCN1 acts as a miR‐24‐3p target in miR‐24‐3p‐mediated sensitivity to PTX.^17^ In this study, the tumour growth in mice was more suppressed in CT‐genotyped tumours compared with the CC‐genotyped tumours, whereas the knockdown of lncRNA‐CCAT1 partly blocked the effects of the CT genotype.

Furthermore, compared with the rs67085638‐CC mice, the levels of lncRNA‐CCAT1 and FSCN1 mRNA/protein were increased whereas the miR‐24‐3p level was decreased in the rs67085638‐CT + NC shRNA mice, and the knockdown of lncRNA‐CCAT1 partly reversed the dysregulation of these genes.

## 5 CONCLUSION

The findings of this study demonstrated that the presence of the minor allele of rs67085638 increased the expression of CCAT1 and enhanced the chemoresistance to PTX, whereas the down‐regulation of CCAT1 partially, but significantly, re‐stored the sensitivity of colon cancer cells to PTX.

## AUTHOR CONTRIBUTION

**Zhong‐sheng Xiao:** Conceptualization (equal); Formal analysis (equal); Investigation (equal); Project administration (equal); Supervision (equal); Writing‐original draft (equal). **Lei Zhao:** Conceptualization (equal); Investigation (equal); Methodology (equal); Software (equal); Validation (equal). **Xiao‐ning Zhang:** Formal analysis (equal); Investigation (equal); Software (equal). **Han‐xian Li:** Investigation (equal); Software (equal); Visualization (equal). **Zhi‐hui Yin:** Methodology (equal); Supervision (equal); Validation (equal); Writing‐original draft (equal); Writing‐review & editing (equal).

## ETHICAL APPROVAL

The Human Research Ethics Committees of The First Affiliated Hospital of University of South China has approved this research, and all methods were performed in accordance with the last vision of the Declaration of Helsinki. Written informed consent was obtained from all patients or their first‐degree relatives before the surgery. All animal experiments were performed in line with the Guide for the Care and Use of Laboratory Animal by International Committees and were approved by the Animal Ethics Committee of The First Affiliated Hospital of University of South China.

## Data Availability

The data that support the findings of this study are available from the corresponding author upon.
